# Nab-Paclitaxel-Based Systemic Approach to Achieving Complete Remission for Relapsed Stage III Endometrial Carcinoma: Insights From the Indian Subcontinent

**DOI:** 10.7759/cureus.57111

**Published:** 2024-03-28

**Authors:** Prasanna Rammohan, Vipulkumar Thummar, Priya Mehta

**Affiliations:** 1 Oncology, Cancure Cancer Centre, Tiruchirappalli, IND; 2 Medical Affairs, Zydus Lifesciences Ltd., Ahmedabad, IND

**Keywords:** nab-paclitaxel, systemic therapy, high risk, relapse, endometrial cancer

## Abstract

Endometrial adenocarcinoma (EC) is a common type of cancer in women that starts in the lining of the uterus. It usually affects women following menopause but can also occur in younger women. There are different types of EC based on how the cells look under a microscope and their molecular characteristics. EC is generally divided into two main groups: Type I, which is linked to estrogen and mainly consists of low-grade cells, is more common and usually has a better outcome; and Type II, which is not related to estrogen, consists of high-grade cells, is less common, and typically has a worse prognosis. This case report presents a comprehensive examination of the clinical course of a 65-year-old female patient who achieved complete remission following a relapse of high-risk EC through a unique therapeutic approach involving Taxonab 300mg/carboplatin 450mg. The case report underscores the significance of investigating nab-paclitaxel-based interventions as a promising strategy for improving outcomes in patients facing the challenging scenario of inoperable, high-risk EC relapses.

## Introduction

Endometrial adenocarcinoma (EC), is a gynecological malignancy characterized by the uncontrolled proliferation of glandular epithelial cells within the endometrial lining and is one of the most common gynecologic cancers affecting women worldwide [1]. EC has an incidence rate of 2.3/100,000 women in India and every year around 400,000 new cases are reported globally [2,3]. It is a heterogeneous group of malignancies that typically manifests with abnormal uterine bleeding, however, the initial presentation can be misleading [1].

In well-differentiated EC, glandular complexities such as luminal infolding, branching, and cribriform structures are observed. In high-grade tumors, increased nuclear atypia, pleomorphism, and irregular chromatin clustering are also observed [4]. The primary treatment modalities for EC in relapsed cases, typically include radical surgery along with lymph node dissection or radical radiotherapy and still have a five-year overall survival of less than 20% [5].

This case report presents the therapeutic trajectory of a patient in their 60s who achieved complete remission from recurrent inoperable stage III endometrial carcinoma. The resolution ensued following the administration of a pioneering neoadjuvant therapy regimen, comprising Taxonab, a novel albumin-bound nanoparticle formulation of paclitaxel that is free of solvents.

## Case presentation

A 65-year-old female patient, post-menopausal, with a known diagnosis of endometrial cancer (International Federation of Gynecology and Obstetrics (FIGO) Stage IB - Grade II) presented to the medical oncology department, with the chief complaint of pelvic pain and lower limb edema in December 2022. The patient was earlier diagnosed with EC in November 2017, when the patient had presented with the chief complaint of chronic cervicitis which was refractory to treatment, indicating further clinical evaluation. Although the patient had no family history of malignancy, histopathological analysis revealed intricate glandular and papillary structures lined with columnar epithelial cells displaying moderate nuclear atypia in the uterine cervix and both tubes. Focal squamous metaplasia, lymph vascular invasion along with infiltration into 3/4th of myometrium was present, which led to the diagnosis of Grade II EC for which the patient underwent vaginal hysterectomy.

**Figure 1:**
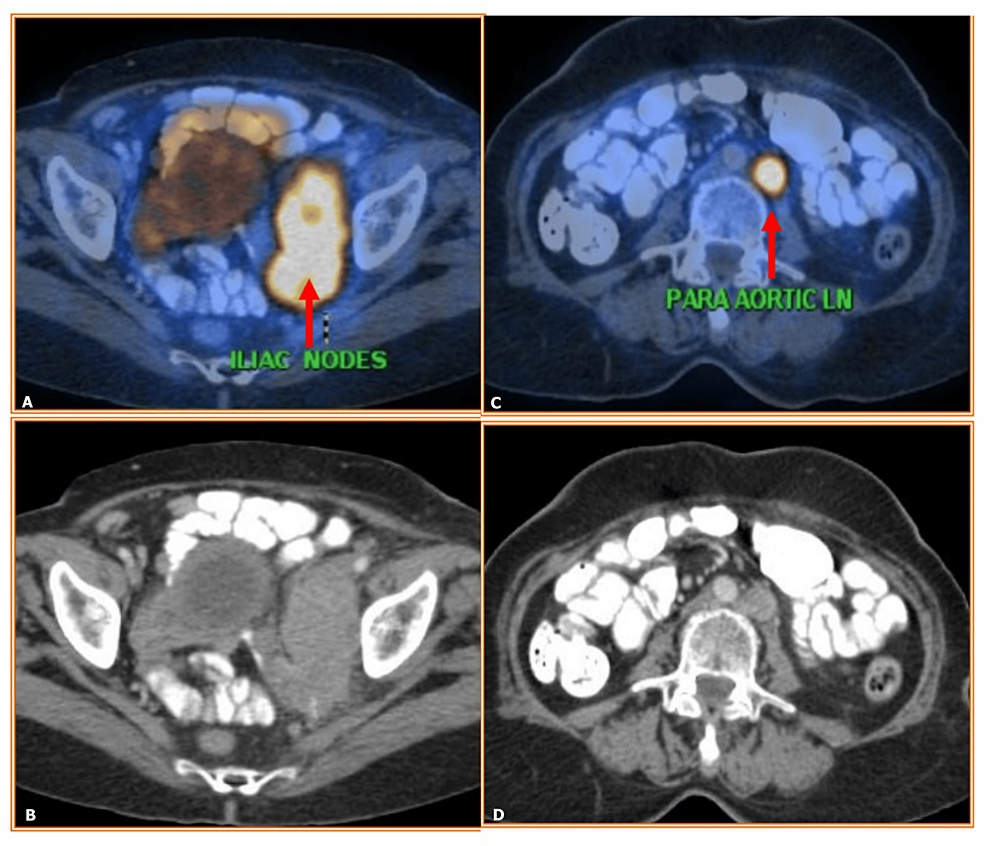
Pre-treatment PET-CT showing (A and B) multiple conglomerated iliac nodes with infiltration to iliac veins (52X42mm) SUVmax 13.6 and (C and D) enlarged left lower para-aortic nodes (16x14mm), SUVmax 12 (red arrows).

Considering the patient's history, ultrasonography (USG) was carried out, revealing a few enlarged left iliac lymph nodes measuring 57x44 mm with areas of necrosis. The PET-CT evaluation showed multiple conglomerated iliac nodes 5.2x4.2cm standard uptake volume (SUV) (13.6) compressing/infiltrating iliac veins; left lower para-aortic node 1.6x1.4cm SUV (12). The molecular profiling analysis with immunohistochemistry (IHC) showed positive POLE (DNA polymerase epsilon, catalytic subunit) gene mutation, negative for P53 wild-type mutation, and microsatellite instability (MSI). Hormone receptor analysis (ER/PR) with IHC showed negative results (Figure 1).

On the basis of these clinical findings, a confirmatory diagnosis of locoregional recurrent endometrial cancer with pelvic/para-aortic lymph nodes (LN) relapse was made. The patient was staged as FIGO stage IIIC EC, which was inoperable, indicating a poor prognosis. Due to the inoperable nature of the EC, the treatment plan included neoadjuvant chemotherapy (NACT) comprising Taxonab 300mg/carboplatin 450mg.

**Figure 2:**
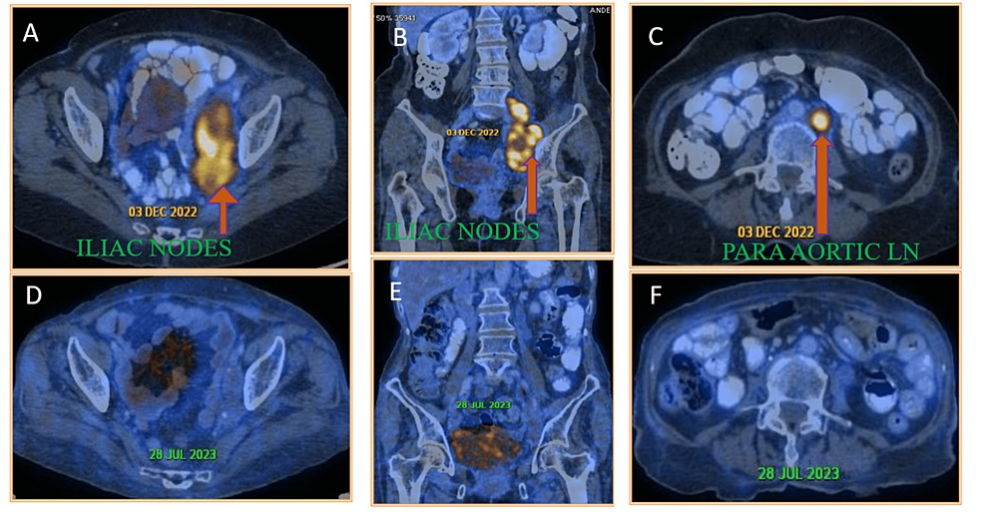
(A, B, C) Post-treatment PET-CT showing significant reduction in iliac nodes (40x15mm) with low SUV uptake (2.3). (D, E, F) Complete resolution of left lower para-aortic nodes.

After two cycles of NACT, the patient showed significant clinical improvement in venous edema. Post three cycles of NACT, the patient was administered pelvic radical chemoradiation of 45 Gy/1.8Gy per fraction over five weeks followed by a 5.4Gy boost to the para-aortic node. Oncological follow-up was carried out and the patient underwent PET-CT assessment two months after the completion of therapy which showed a marked reduction in iliac LN size to 4cmx1.5cm SUV (2.3) and near complete resolution of left para-aortic LN (Figure 2).

## Discussion

Endometrial carcinoma poses a notable clinical challenge upon recurrence in a locoregional manner. The duration of relapse typically spans three years following radical treatment, with long-term recurrences, particularly in radiotherapy-naïve cases, being possible but uncommon [1,6]. The patient in the current case who was earlier diagnosed with stage II EC had experienced a relapse five years post-vaginal hysterectomy. The lesion was diagnosed as FIGO stage IIIC2 EC. The EC staging recommendations as per the American Joint Committee on Cancer (AJCC) and FIGO have defined stage II as cancer encompassing tumors with cervical stromal invasion. Stage IIIC is further divided into C1 (involving pelvic lymph node metastasis) and C2 (involving para-aortic lymph node metastasis). The patient underwent molecular analysis with immunohistochemistry, depicting positive POLE gene mutation and negative wild-type P53 and MSI. The presence of POLE demonstrates favorable prognostic possibilities, despite rare and high-grade tumours [7,8].

The National Comprehensive Cancer Network (NCCN) guidelines for locoregional recurrence in endometrial cancer include surgical exploration or radiation therapy at the site of recurrence. However, as in the present case in which the patient was radiotherapy (RT)-naive and at an inoperable IIIC stage, NCCN guidelines are not specific, creating a dilemma for clinicians [9]. The treatment guidelines for cases of locoregional recurrence of EC following a hysterectomy are poorly defined, leaving clinicians with a lack of specific recommendations [10,11]. Also, the conventional treatment approach for advanced EC historically involved a combination of chemotherapy drugs, namely carboplatin and paclitaxel. This regimen is effective in approximately 50% of patients, although the majority experience disease progression within one year [3].

The treatment regimen in the present case comprises nab-paclitaxel 300mg/ carboplatin 450mg as a NACT prior to pelvic external beam radiotherapy (EBRT), which is a unique approach to treating inoperable stage IIIC EC. Nanoparticle albumin-bound paclitaxel (nab-paclitaxel, Taxonab), was developed to address the clinical shortcomings such as increased hypersensitivity reactions (HSR) related to paclitaxel administration. Taxonab is a new-generation target agent that uses nanotechnology to produce solvent-free gelatinous particles, rendering higher effectivity, minimizing HSR occurrence, and reducing systemic toxicities such as peripheral sensory neuropathy and myelosuppression [12,13].

In a phase II study, nab-paclitaxel was administered in patients who experienced relapse after a prior cytotoxic regimen for metastatic cervical cancer at the dose of 125 mg/m2 on Days 1, 8, and 15 every 28 days. Faced with such undifferentiated malignancy characterized by aberrant angiogenesis, this therapeutic regimen demonstrated robust control in minimizing systemic toxicity, further validating the use of this unique therapy in relapsed high-grade cancers [14]. The nab-paclitaxel formulation is also readily absorbed by both tumor cells and immune cells. Once absorbed, notable immunostimulatory effects that enhance the cancer-immunity cycle are observed [15].

 A Japanese open-label, phase III GOG-99 (Gynaecologic Oncology Group), and the PORTEC-1, -2, and -3 (Postoperative Radiation Therapy in Endometrial Cancer) trial demonstrated that RT was superior to three cycles of paclitaxel and carboplatin chemotherapy in high and intermediate risk EC [16]; however, the present case, due to its inoperable stage IIIC phase, was treated with NACT of nab-paclitaxel 300mg followed by pelvic/para-aortic RT that showed complete resolution, underscoring the exceptional efficacy of nab-paclitaxel-based NACT as a potential standalone approach.

Thus, the use of nab-paclitaxel plus carboplatin in combination with EBRT and RT for EC is gaining attention as an innovative approach in the management of advanced and inoperable cases. A corresponding study conducted by Wang et al. (2023) demonstrated that nab-paclitaxel combined with cisplatin for advanced uterine cancer showed unprecedented improvements in terms of safety and efficacy compared to patients receiving only paclitaxel [12]. This case thus underscores that tailored strategies for individualized management of high-grade tumors can yield remarkable results.

## Conclusions

The combination of nab-paclitaxel-based NACT along with RT offers a promising approach to achieving significant control in inoperable recurrent cases. This case report provides valuable insights into the evolving landscape of EC management, highlighting the potential for improved outcomes through tailored treatment strategies. Further research and clinical trials are warranted to validate the efficacy and long-term benefits of this approach in a larger patient population.
